# In *vitro* impact of pegvisomant on growth hormone-secreting pituitary adenoma cells

**DOI:** 10.1530/ERC-16-0140

**Published:** 2016-07-01

**Authors:** Thomas Cuny, Caroline Zeiller, Martin Bidlingmaier, Céline Défilles, Catherine Roche, Marie-Pierre Blanchard, Marily Theodoropoulou, Thomas Graillon, Morgane Pertuit, Dominique Figarella-Branger, Alain Enjalbert, Thierry Brue, Anne Barlier

**Affiliations:** 1 Aix-Marseille UniversityCNRS, CRN2M UMR7286, Marseille, France; 2 Endocrine Research UnitMedizinische Klinik und Poliklinik IV, Klinikum der LMU, Munich, Germany; 3 APHM TimoneDepartment of Neurosurgery, Marseille, France; 4 APHMConception, Laboratory of Molecular Biology, Marseille, France; 5 Department of EndocrinologyMax Planck Institute of Psychiatry, Munich, Germany; 6 APHM TimoneLaboratory of Neuropathology and Aix-Marseille University, INSERM, CRO2 UMR911, Marseille, France; 7 APHM ConceptionDepartment of Endocrinology, Marseille, France

**Keywords:** acromegaly, pegvisomant, prolactin, GH4C1, pituitary adenoma

## Abstract

Pegvisomant (PEG), an antagonist of growth hormone (GH)-receptor (GHR), normalizes insulin-like growth factor 1 (IGF1) oversecretion in most acromegalic patients unresponsive to somatostatin analogs (SSAs) and/or uncontrolled by transsphenoidal surgery. The residual GH-secreting tumor is therefore exposed to the action of circulating PEG. However, the biological effect of PEG at the pituitary level remains unknown. To assess the impact of PEG *in vitro* on the hormonal secretion (GH and prolactin (PRL)), proliferation and cellular viability of eight human GH-secreting tumors in primary cultures and of the rat somatolactotroph cell line GH4C1. We found that the mRNA expression levels of GHR were characterized in 31 human GH-secreting adenomas (0.086 copy/copy β-Gus) and the GHR was identified by immunocytochemistry staining. In 5/8 adenomas, a dose-dependent inhibition of GH secretion was observed under PEG with a maximum of 38.2±17% at 1μg/mL (*P*<0.0001 vs control). A dose-dependent inhibition of PRL secretion occurred in three mixed GH/PRL adenomas under PEG with a maximum of 52.8±11.5% at 10μg/mL (*P*<0.0001 vs control). No impact on proliferation of either human primary tumors or GH4C1 cell line was observed. We conclude that PEG inhibits the secretion of GH and PRL in primary cultures of human GH(/PRL)-secreting pituitary adenomas without effect on cell viability or cell proliferation.

## Introduction

Acromegaly is a rare disease with a prevalence of around 40–130 cases per million inhabitants (Chanson *et al.* 2014). It is caused by oversecretion of the growth hormone (GH), mainly by a pituitary GH-secreting adenoma (Chanson *et al.* 2014). Treatment of GH-secreting adenomas consists primarily of transsphenoidal surgery followed by a personalized medical therapeutic approach (whose indications can be discussed at every step of the therapeutic strategy) and in some cases radiotherapy (Giustina *et al.* 2014, Katznelson *et al.* 2014). Somatostatin analogs (SSAs) with long-acting release (octreotide and lanreotide) represent medical therapies of choice in acromegaly to control GH and IGF1 oversecretion because these agents specifically bind to the somatostatin receptor subtype 2, a membrane receptor highly expressed in somatotroph cells and whose activation leads to both antisecretory and antiproliferative effects (Chanson 2015). Their efficacy varies from one patient to another: a recent meta-analysis reported an overall control rate of 56% for mean GH and 55% for IGF1 normalization in SSA-treated patients (Carmichael *et al.* 2014). Patients totally or partially resistant to SSA are therefore good candidates for the GH-receptor (GHR) antagonist pegvisomant (PEG), administered alone or in combination with a SSA and/or dopamine agonists (Neggers *et al.* 2016). The exon 3 deleted GHR isoform (d3-GHR) is associated with better response to PEG therapy in acromegaly (Bernabeu *et al.* 2010). This isoform results from a homologous recombination of two almost identical retroelements flanking exon 3 that produce a 2.7kb deletion, giving rise to two different isoforms of GHR (full-length (fl-GHR) and d3-GHR).

PEG is a genetically engineered and pegylated analog of human GH developed in the late 1980s, which functions as a selective GHR antagonist thereby inhibiting the synthesis and release of IGF1 by the liver by abrogating the STAT5-mediated GHR signalization (Kopchick *et al.* 2002). PEG binds to a preformed GHR dimer, but without binding properly the site 2 necessary to induce intracellular GHR signaling (Chen *et al.* 1991).

From a clinical perspective, a majority of acromegalic patients achieve a normalization of IGF1 levels in controlled clinical trials when an optimal dose titration of PEG is insured at successive endpoints (van der Lely *et al.* 2001). Clinical studies in daily practice rather report that around 70% of patients are biochemically controlled by PEG, although with a significant impact on acromegalic comorbidities (Kuhn *et al.* 2015).

Although the peripheral effect of PEG on different tissues (lung, breast and colon) has been well studied (Kopchick *et al.* 2002), little is known about its impact at the pituitary level, especially on the normal and tumoral GH cells. After injection, PEG may reach the pituitary gland and, possibly, any residual unresectable GH-secreting tumor via the bloodstream. As GHR is expressed in both normal and tumoral somatotroph cells (Mertani *et al.* 1995, Kola *et al.* 2003, Beuschlein *et al.* 2005) and patients treated with PEG display an increase in circulating endogenous GH (van der Lely *et al.* 2001), questions have arisen about the possibility that PEG could promote growth of residual tumors either directly via GHR or indirectly through the increase in endogenous GH.

The purpose of this study was to investigate the effect of PEG *in vitro* both on hormonal secretion and cellular viability of both primary cultures of human GH-secreting pituitary adenomas and of the rat somatolactotroph GH4C1 cell line.

## Design and methods

### Patients

This *in vitro* study included somatotroph tumors from patients undergoing transsphenoidal surgery at the Neurosurgery Department of Marseille and Munich hospitals. This study was approved by the Ethics Committee of the University of Aix-Marseille II (Aix-Marseille, France) and informed consent was obtained from each patient. The endocrine and neuroradiological characteristics of the tumor were documented before any treatment. All tumor fragments were subjected to anatomopathology analysis that confirmed the GH or GH/PRL phenotype by immunohistochemistry. Thirty-one somatotroph tumors were included in this study. Among them, eight were used for *in vitro* experiments (Table 1). Three patients (cases 3, 4, and 6) were pretreated with SSA before finally undergoing surgery.

**Table 1:** Clinical and *in vitro* characteristics of the eight human GH-secreting tumors.

				**Hormone secretion** (*in vivo*)			**Hormone secretion** (*in vitro*)*				
**Case no**	**Sex**	**Age (year)**	**Tumor size (mm)**	**GH (ng/mL)**	**IGF1 (ng/mL) (normal range)**	**PRL level (ng/mL) (*N*<15 (male) <25 (female))**	**GH (ng/mL)**	**PRL (ng/mL)**	**Max GH inhibition (%) under PEG**	**Max PRL inhibition (%) under PEG**	**GHR mRNA level (copy/copy β-Gus)**
1**	M	55	Macro (19)	1.5	**404** (54.6–185.7)	189	54	49	27	54	0.219
2	F	23	Macro (NA)	26.6	**993** (149.1–332.3)	60	28	Un	65	NA	0.407
3	M	26	Macro (NA)	2.4	**1105** (96.4–227.8)	NA	404	Un	15***	NA	0.01
4	F	79	Macro (20)	5.3	**1352** (54–204.4)	25	312	Un	39	NA	0.020
5**	F	45	Macro (NA)	8.3	**804** (92.7–244.6)	72	12	38	12***	33	0.085
6	F	59	Macro (16)	8.8	**718** (54–204.4)	10	276	Un	0***	NA	0.015
7**	M	56	Macro (20)	3.3	**711** (54.6–185.7)	102	385	45	31	46	0.476
8	F	59	Micro (8)	3	**930** (54–204.4)	10	310	Un	24	NA	0.185

* GH and PRL release by 50×10^3^ cells cultured during 72h, expressed as nanograms in 1mL of culture medium

** Cases 1, 5, and 7 were mixed GH/PRL adenomas

*** Cases 3, 5, and 6 were considered as non-responsive to pegvisomant in our work when considering inhibition of GH secretion (maximal GH inhibition ≤15%).

NA, not available; Un, undetectable.

### Pharmacological compounds

PEG was provided by Pfizer and used at concentrations ranging from 0.1 to 10μg/mL. The range of concentrations of PEG required to antagonize GHR on the somatotroph cell was arbitrarily established by considering the endogenous GH secretion released in the medium from 20,000 GH adenoma cells over an 8-h period (Saveanu *et al.* 2002). Octreotide was a kind gift of Novartis International AG (Basel, Switzerland).

### Cell culture

#### Primary culture of human pituitary adenomas

Tumor fragments obtained from transsphenoidal surgery were submitted to mechanical and enzymatic dissociation with collagenase at 37°C for 60min. Total cell amounts were 4×10^6^ to 65×10^6^, depending on the tumor. Antifibroblast microbeads (Anti-Fibroblast Microbeads, human; Miltenyi Biotec, Paris, France) were used to eliminate fibroblasts, according to the manufacturer’s protocol. Adenoma cells were plated on well dishes or glass coverslips, coated with extracellular matrix of bovine corneal epithelial cells (ECM) as described previously (Jaquet *et al.* 1985). For hormonal assays, tumor cells were plated at a density of 50×10^3^ cells in 24-well cultures dishes. The cells were cultured in appropriate DMEM, depleted in l-valine (d-valine was indeed used instead of l-valine to block fibroblast proliferation), and supplemented with 10% fetal calf serum (FCS), penicillin (100U/mL), streptomycin (100U/mL), and glutamine (100U/mL) and maintained at 37°C in an atmosphere containing 7% CO_2_. They were then washed and the medium replaced by d-valine DMEM containing 1% FCS referred to hereafter as ‘low serum medium’ (Jaquet *et al.* 1985).

#### Culture of the rat somatolactotroph cell line GH4C1

The rat somatolactotroph GH4C1 cell line (ATCC CCL-82.2, USA) was grown in Ham’s F-10 medium supplemented with 15% horse serum (Eurobio, France), 2.5% FCS, penicillin (50U/mL), and streptomycin (50μg/mL) and was maintained at 37°C in an atmosphere of 7% CO_2_. Cells were subcultured weekly and the medium was changed twice a week.

### Determination of GHR mRNA levels by real-time PCR

Total mRNAs were extracted from somatotroph fragments using the RNeasy Mini kit (Qiagen, Cat. no. 74104) and from GH4C1 cell pellets using the RNeasy Micro kit (Qiagen, Cat. no. 74004). One microgram of total RNA was used for cDNA synthesis during reverse transcription using the First-Strand cDNA Synthesis Kit (GE Healthcare Life Sciences). Human GHR mRNA was detected by real-time quantitative PCR using TaqMan Gene Expression Assay (Applied Biosystems, Assay ID Hs00174872_m1 and Hs00168739_m1) and rat GHR (rGHR), using Rn_Ghr_1_SG primer assay (Qiagen). The human GHR mRNA levels were normalized to the beta-glucuronidase (β-Gus) mRNA level and rGHR to the rat β-actin mRNA level. Standard curves were drawn using dilution plasmid gamma verified by PCR and linearized.

#### Somatic analysis of human GHR sequence

Genomic DNA from 21 of the 31 human somatotroph tumors was extracted and the coding exons and exon–intron boundaries of the GHR gene were screened by a direct PCR sequencing. A multiplex PCR procedure was performed to identify fl-GHR and d3-GHR alleles, as described previously (Pantel *et al.* 2000).

#### Immunocytochemistry of GHR and rGHR

The expression and localization of the human and rat GHR were assessed by immunocytochemistry on human somatotroph cells and GH4C1, respectively. Human cells were grown on 14-mm extracellular matrix-coated glass coverslips and GH4C1 on 14-mm poly-lysine-coated glass coverslips. After fixing, the cells were incubated overnight at 4°C with an anti-GHR antibody (Abcam ab78426, Abcam) diluted to 1/100 in PBS supplemented with 1% bovine serum albumin (Sigma). The immunostaining was visualized using Alexa 488-conjugated goat antirabbit IgG (Molecular Probes, Invitrogen) diluted to 1/800 in PBS containing 10% normal goat serum. The nucleus of each cell was visualized through DAPI counterstain (in blue). Confocal images were acquired on a Zeiss LSM780 laser-scanning microscope and the image editing was performed using Adobe Photoshop. To quantify GHR immunostaining, gray-scale images were adjusted with a common minimum and maximum threshold, and the integrated density was measured using ImageJ (1.40g software).

### PEG pharmacological studies

For human tumoral somatotroph cells, 2×10^4^ cells were incubated in low serum medium with or without increasing doses of PEG for 3days. Each experimental condition was assayed in triplicate wells. After 3days, cell viability was assessed by a luminescent cell viability assay (CellTiter-Glo, Promega) according to the manufacturer’s protocol. Supernatants were collected, clarified by centrifugation at 400***g>***, and frozen before measuring hormonal levels.

For bromodeoxyuridine (BrdU) incorporation, 5×10^3^ human tumoral cells were plated in a 24-well plate. After 24h, the cells were incubated in a low serum medium and treated or not with increasing doses of PEG for 3days. On the third day, BrdU was added to a final concentration of 1μM. After incubation for 16h, DNA synthesis was assayed with the Cell Proliferation ELISA BrdU (Roche Molecular Biochemicals, Meylan, France). The newly synthesized BrdU-DNA was determined using a microplate reader (Berthold Technologies, Thoiry, Yvelines, France).

For GH4C1, 2.5×10^4^ cells were plated in 24-well dishes for 48h before medium removal and replacement with appropriate low serum medium (5% horse serum (HS), 0.8% FCS), with or without PEG. Cell viability was then assessed by luminescence assay (CellTiter-Glo).

For 5-ethynyl-2′-deoxyuridine (EdU) (i.e. a BrdU analog) incorporation analysis, 10^4^ GH4C1 cells were plated in a black 96-well plate. After 24h, the cells were incubated in low serum medium and treated with or without the drug for 3days. Each day, EdU was added to a final concentration of 10μM. After incubation for 16h, DNA synthesis was assayed with the Click-iT EdU Alexa Fluor 647 HCS Assay (Invitrogen, Molecular Probes). The newly synthesized EdU-DNA was determined using a microplate reader (Berthold Technologies, Thoiry, Yvelines, France) and reported to cell number (incorporation/cell number ratio) determined by HCS Nuclear Mask blue staining.

### Hormonal assays

Endogenous GH was assayed using a PEG-insensitive based two-site immunoassay utilizing specific monoclonal antibodies as described previously (Veldhuis *et al.* 2010, Manolopoulou *et al.* 2012). The assay was calibrated against 22-kDa recombinant human GH (International Reference Preparation 98/574). To adjust for matrix differences, standard curves were prepared in the respective cell culture media. In this setting, intra- and inter-assay variabilities were below 4.8 and 9.2%, respectively.

Endogenous PRL levels were measured using a commercial PRL IRMA kit (PRL IRMA Kit, Beckman Coulter Immunotech, Marseille, France) with a detection limit above 1ng/mL. The coefficients of intra- and inter-assay variations were less than or equal to 2.8 and 8%, respectively.

### GHR pathway analysis by western blot

The JAK2 and STAT5 activation levels were assessed by western blot analysis on human somatotroph tumoral cells (Romano *et al.* 2003). Briefly, 1×10^6^ cells were plated in 6-well dishes and incubated with 10μg/mL PEG for 5min in low serum medium. Then, total denatured proteins were separated on 10% SDS-PAGE gels and transferred to polyvinylidene diﬂuoride membranes (PerkinElmer). Immunodetection of JAK2 was performed using a JAK2 rabbit monoclonal-speciﬁc antibody (1:1000; D2E12; Cell Signaling Technology) and the phosphorylated form of JAK2 on tyrosine residues 1007/1008 was immunodetected by a specific phospho-JAK2 monoclonal antibody (1:1000, C80C3, Cell Signaling Technology). Immunodetection of both STAT5 and phospho-STAT5 was performed using a STAT5 rabbit monoclonal antibody (1:1000; 3H7; Cell Signaling Technology) and a rabbit monoclonal antibody able to bind to phosphorylated residue tyrosine 694 of STAT5 (1:1000; C71E5; Cell Signaling Technology). Blots were developed with the enhanced chemiluminescence CDP-Star detection system (Applied Biosystems) and quantified with a GBox (Ozyme, France).

### Statistical analysis

GHR mRNA levels were presented as the median and hormonal results as the mean±s.e.m. Statistical significance between groups was determined by the ANOVA Tukey’s multiple-comparison test. A *P* value <0.05 was considered to be significant for all tests. To measure the strength of association between pairs of variables without specifying dependency, Spearman’s rank-order correlations were run. The differences were considered to be statistically significant at *P*<0.05.

## Results

### Characterization of the GHR in human somatotroph tumors

From our series of 21 human somatotroph tumors, no mutations of GHR were found, including the p.His49Lys mutant known to impair GHR function (Asa *et al.* 2007). Two tumors (2/21, 9.5%) were homozygous for the d3-GHR isoform, a roughly similar rate to that observed in the study conducted by Pantel *et al*. (2000).

**Figure 1:**
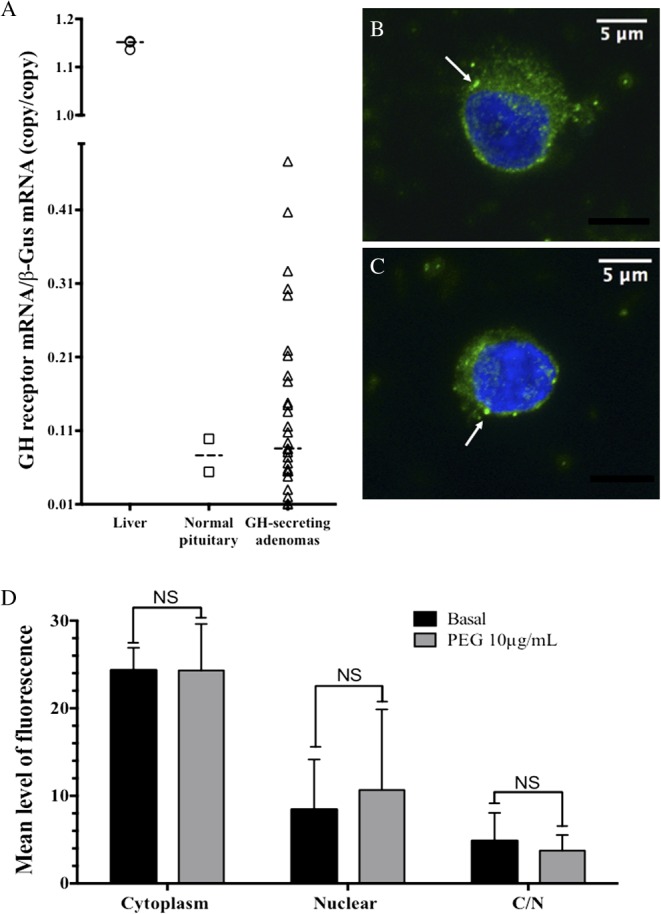
Expression of the GHR in human GH-secreting adenomas. (A) mRNA expression of GHR assessed by real-time PCR in human liver (*n*=3), normal pituitary tissue (*n*=2), and GH-secreting pituitary adenomas (*n*=31). Note that 18 out of 31 adenomas display a higher mRNA level of GHR than the normal pituitary tissue. (B and C) GHR immunostaining as described in the ‘Design and methods’ section in a representative primary culture of a GH-secreting adenoma (63×, zoom factor 1) at T0 (B) and at T24 (hours) of pegvisomant 10μg/mL (C). Nucleus is stained in blue (DAPI) and GHR is shown as green spots (arrows). (D) Mean quantification of fluorescence of the GHR in both the cytoplasm and the nuclear compartment of GH-secreting cells from the tumor case no. 8. C/N, cytoplasm/nuclear ratio.

Overall, 31 human GH-secreting tumors were analyzed for the GHR mRNA expression. Among them, eight were subjected to *in vitro* pharmacological studies: five pure somatotroph and three somatolactotroph adenomas (Table 1). GHR expression was found in all the tested tumors, with a median of 0.086 copy/copy β-Gus and most of them (*n*=18) displaying a higher expression of GHR than that found in the normal pituitary (*n*=2, 0.076 copy/copyβGus), although markedly less than the expression observed in the liver (*n*=3, 1.15 copy/copy β-Gus) (Fig. 1A).

Once the endogenous GH is bound, a conformational switch for the GHR occurs with a subsequent translocation within the nuclear compartment (Conway-Campbell *et al.* 2007). We assessed the subcellular localization of the GHR by immunofluorescence in human somatotroph cells both in basal (Fig. 1B) and PEG-treated conditions (Fig. 1C). The GHR immunolabeling showed predominant cytosolic spots in basal conditions without significant change of the labeling profile after 24h of PEG 10μg/mL (Fig. 1D).

### Impact of PEG on cellular viability, hormonal secretion, sensitivity to octreotide and GHR activation of human somatotroph adenomas

**Figure 2:**
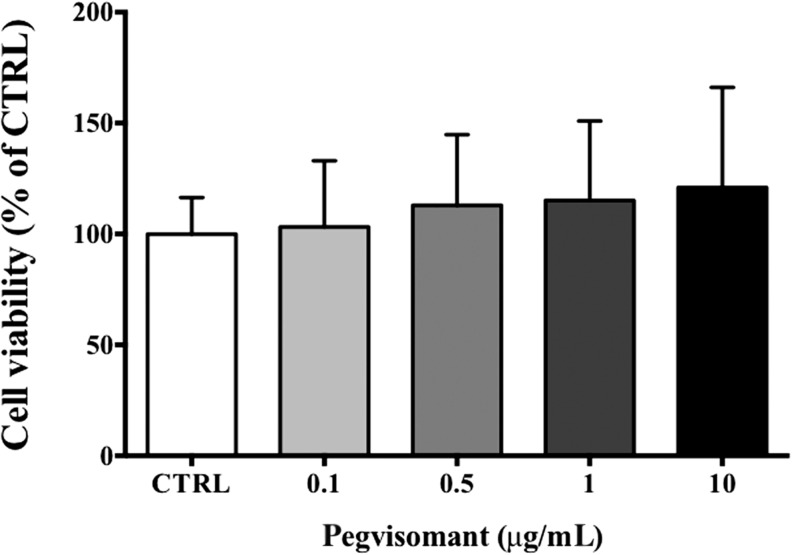
Cell viability of human GH-secreting adenomas (*n*=6) in primary culture. Cell viability was assessed by CellTiter-Glo (see ‘Design and methods’ section) under increasing doses of PEG ranging from 0.1 to 10μg/mL and measured after 72h of treatment. CTRL, control. Because of an insufficient amount of cells collected after dissection, tumors of cases 1 and 5 have not been included in this experiment.

No impact of PEG was observed over the 72-h treatment period either on cellular viability (Fig. 2) or incorporation of BrdU (data not shown) in the six tested tumors whatever the concentration used.

**Figure 3:**
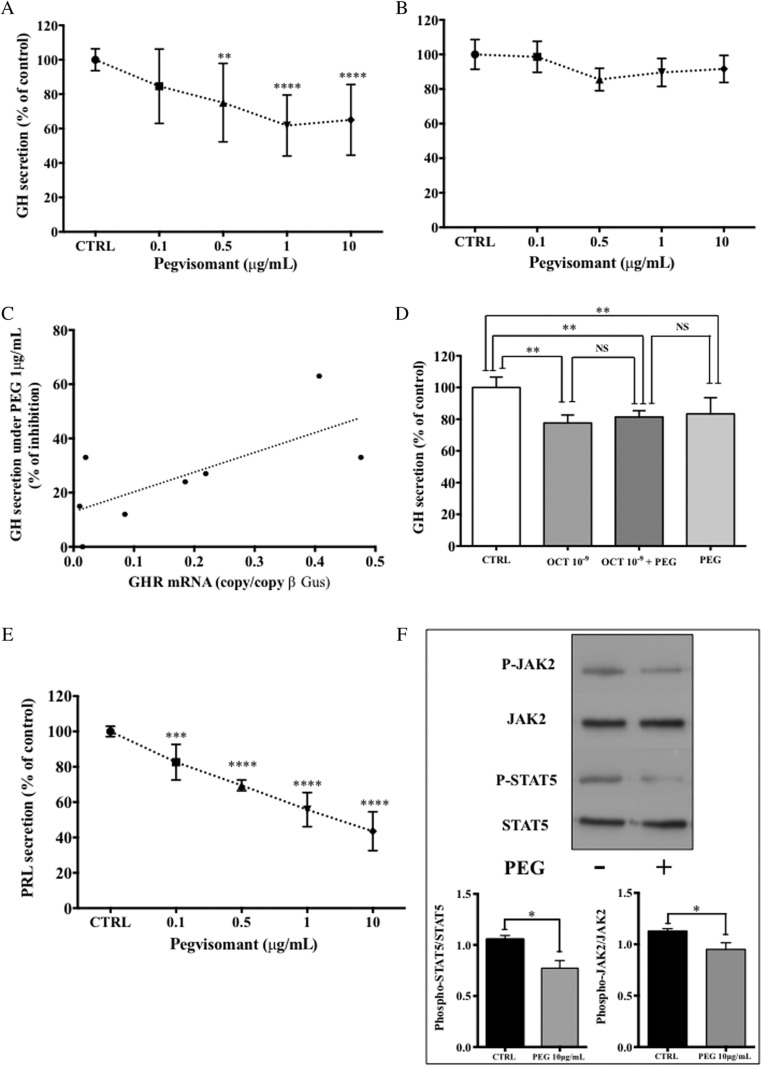
Effect of PEG on hormonal secretions of primary cultures of human pituitary adenomas. (A and B) Mean dose–response GH suppression obtained in cell cultures from eight GH-secreting adenomas (Table 1) under PEG (0.1–10 μg/mL) categorized as five PEG-responsive tumors (A) (****P < 0.0001 vs CTRL) and three PEG-non-responsive tumors (B). (C) Correlation between maximal inhibition of GH secretion under PEG 1 μg/mL and GHR mRNA expression levels in eight GH-secreting adenomas (*r* = 0.68, *P *= 0.07). (D) Inhibition of GH secretion in two secreting adenomas (cases 1 and 4) after 72 h of octreotide 10^–9^ mol/L alone (OCT 10^–9^), PEG alone 10 μg/mL (PEG), or combination of both (OCT 10^–9 ^π PEG). **P < 0.01. (E) Mean dose–response of PRL suppression obtained in cell cultures from three mixed GH/PRL adenomas (cases 1, 5, and 7) under PEG (0.1–10 μg/mL) ***P < 0.001, ****P < 0.0001 vs CTRL. (F) Western blot analysis of phospho-STAT5/STAT5 and phospho-JAK2/JAK2 in two GH-secreting adenomas (cases 2 and 8) after 5 min of treatment by PEG (10 μg/mL). Upper panel: representative blot of one tumor (case 2). Lower panel: quantification of both phospho-STAT5/STAT5 and phospho-JAK2/JAK2 on the immunoblot of the two tumors. *P < 0.05 vs CTRL.

Moreover, PEG did not induce an increase in GH secretion in the eight tested tumors. Conversely in five of the eight tumors tested, a dose-dependent inhibition of GH secretion occurred (Fig. 3A). We arbitrarily defined a tumor as being sensitive to PEG when the maximal GH inhibition under PEG was more than 15% compared with controls (Table 1). In those five tumors, the mean maximal inhibitory effect observed with the dose of 1μg/mL reached 38.2±17% (*P*<0.0001 vs CTRL). In the three remaining tumors, PEG did not significantly impact the GH secretion whatever the concentration of the drug used (Fig. 3B). Although the analysis did not reach statistical significance, probably due to the low number of tumors, we observed a trend for a positive correlation between the expression level of GHR mRNA and the percentage of maximal inhibition of GH secretion under PEG 1μg/mL (*r*=0.68, *P*=0.07, Fig. 3C). Insufficient tumoral material was available to screen for the d3-GHR isoform in these tumors. When combined with octreotide (10^–9^mol/L), PEG (10μg/mL) did not result in an additive effect on the inhibition of GH secretion in any of the tumors tested (Fig. 3D).

When considering the PRL secretion in the three mixed GH/PRL adenomas, we also observed dose-dependent inhibition of secretion under PEG with a maximum of 52.8±11.5% (*P*<0.0001 vs control) at 10μg/mL (Fig. 3E).

Because the effect of PEG on both GH and PRL secretion was unexpected, we investigated the effect of PEG on the phosphorylation pattern of the two main proteins involved in the GHR transduction pathway. Accordingly, PEG (10μg/mL) decreased the phosphorylated form of both JAK2 and STAT5 (Fig. 3F).

### Impact of PEG on the cellular viability of the somatolactotroph cell line GH4C1

**Figure 4:**
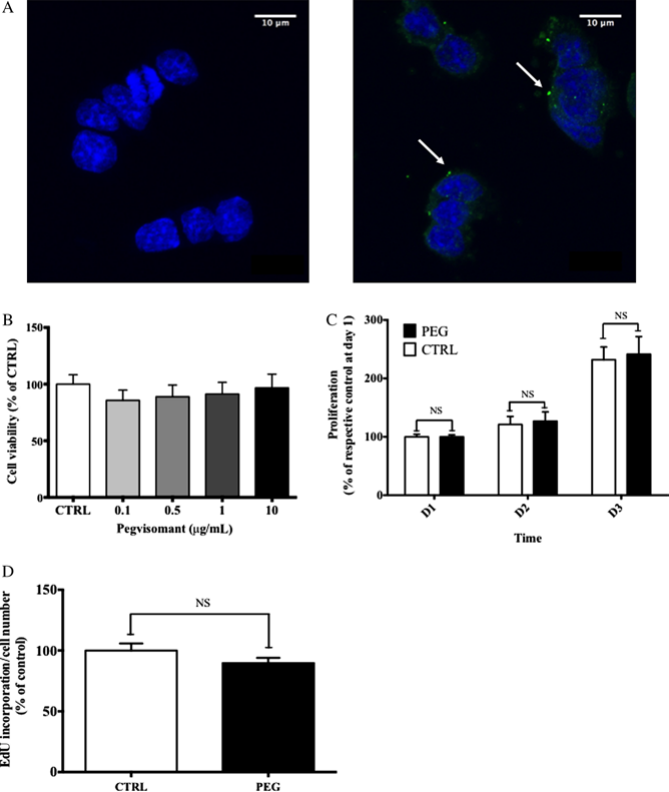
Expression of rat GHR and cell viability and proliferation under pegvisomant in GH4C1 cell line. (A) Confocal microscopy (63×, zoom factor 1). Left panel, control without primary antibody. Right panel, rGHR is labeled in green (arrows) when cells were incubated with the primary (directed against rGHR) and the secondary antibody. Nucleus is stained in blue (DAPI). (B) No effect of 72h PEG (0.1–10μg/mL) on the cell viability of GH4C1 assessed by CellTiter-Glo (*n*=3 experiments). (C) Proliferation of GH4C1 cell lines assessed by EdU incorporation from day 1 to day 3 under PEG 10μg/mL compared with control (CTRL) NS, no significant. (D) Impact of PEG (10μg/mL) on the EdU incorporation/cell number ratio of GH4C1 cell lines after 3days, compared with control (CTRL).

Because human GH-secreting adenoma cells displayed a low proliferation rate in the primary culture, we analyzed both cell viability and proliferation of the somatolactotroph GH4C1 cell line. The GHR was expressed both at the mRNA (0.4 copy/copy actin×10^6^) and at the protein levels in these cells. Similar to the human tumor, the GHR was expressed both in diffuse and spot patterns mainly in the cytosol (Fig. 4A).

No effect on cell viability was observed after 3days of increasing doses of PEG (Fig. 4B). PEG (10μg/mL) had no impact on the proliferative rate of GH4C1 cells in low serum medium from day 1 to day 3 (Fig. 4C). Moreover, the EdU incorporation/cell number ratio was not different after 3days of 10μg/mL PEG (Fig. 4D).

## Discussion

The treatment of acromegaly remains a real challenge in spite of the numerous drugs developed in this field, which have gradually enriched the panel of therapeutic tools (Chanson 2015, Neggers *et al.* 2016). In cases of incomplete surgery and/or resistance to the SSAs, the GHR antagonist PEG, represents a suitable option and rapidly leads to IGF1 normalization in most patients when appropriately titrated. Once injected, PEG does not cross the blood–brain barrier and therefore does not exert a biological effect on the CNS (Veldhuis *et al.* 2010). In contrast, the pituitary gland and any adenoma that has developed within are not ‘protected’ by the blood–brain barrier and are potentially exposed to the circulating PEG if this one can cross the capillary wall in the anterior pituitary. Latter is a *sine qua non* condition whose veracity remains to be determined for suspecting a secondary effect of PEG at the pituitary cell level. Yet, evidences exist to support the idea that (a small amount of) PEG indeed crosses the capillary wall within the anterior pituitary: Nass *et al*. (2000) showed that injection of an analog of PEG in the cerebroventricular space (on the other side of the blood–brain barrier) of rats led to an increase of GH secretion, which suggests a central regulation of GH on its own secretion (negative feedback loop), occurring at the hypothalamic levels where the permeability of capillaries is roughly the same as for those found in the anterior pituitary. Moreover, other bigger molecules than PEG (whose molecular mass is around 40–50kDa) have been recently proposed to potentially cross the capillary wall, like ipilimumab, a recombinant monoclonal antibody of 148kDa directed against cytotoxic T-lymphocyte-associated protein 4 (CTLA-4), which can trigger an hypophysitis state in humans by binding the CTLA-4 when expressed on the pituitary cells (Iwama *et al.* 2014).

In light of the so-called Nelson’s syndrome secondary to bilateral adrenalectomy and abolition of the cortisol-induced negative feedback, the issue concerning the role of PEG in promoting growth of a residual GH-secreting tumor by decreasing the IGF1 plasma concentration has been raised (Marazuela *et al.* 2011) and remains unclear.

Our study focused on the effects of PEG on the secretion and viability of human GH-secreting pituitary adenomas. We then demonstrated that the GHR is expressed both at the mRNA and protein levels in human GH-secreting pituitary adenomas. We then wondered whether PEG might influence the proliferation of GH tumoral cells by binding to the GHR. In our experimental model, PEG does not affect the cell viability of either human or rat somato(lactotroph) tumoral cells; however, two obvious limitations need to be discussed: first, the size of our tumor sample is pretty limited and extrapolating data from our results to establish a general conclusion appears to be anticipated unless other studies confirmed roughly similar results in future works. The second point concerns the eight pituitary tumors, randomly selected in our work and for which the natural history cannot be precisely known until a progression and/or a relapse happen. As a proof-of-concept in clinical trial, our study has the advantage to be the first one to investigate in human GH adenomas primary cultures the impact of PEG at the pituitary level in presence of a functional GH receptor and would hopefully pave the way for further investigations in this direction. It needs to be made clear that even though proliferation may not be the optimal parameter to conclude about a potential PEG effect (because such tumors exhibit a really low rate of proliferation), results we have concerning both the DNA nucleoside incorporation and the GH4C1 experiments strongly suggest that PEG does not modify at all the cellular replication homeostasis even in the short term. This observation aligns closely with the clinical and MRI data originating from the ACROSTUDY, where no significant increase in pituitary size was observed under PEG in 141 acromegalic patients over a median period of 4.9years (Neggers *et al.* 2014). Nevertheless, it must be noted that in this cohort, acromegalic patients were systematically treated with PEG in combination with SSA whose antiproliferative effect may partly obscure the real effect of PEG on tumor mass. Likewise, in the German PEG observational study, 18 of the 307 (5.9%) patients treated with PEG for an average of 86weeks showed an increase in tumor mass, but only eight of these apparent progressions were confirmed after centralized image reevaluation (Buchfelder *et al.* 2009). Throughout the literature, the percentage of patients with authentic tumor growth under PEG therapy remains minimal, between 3 and 6% (Jimenez *et al.* 2008, Buchfelder *et al.* 2009, van der Lely *et al.* 2012). Note that for this small proportion of patients, it is still unclear to distinguish so far a potential proliferative effect of the PEG from the natural history of the tumor. Indeed, most of these patients were candidates to PEG, insufficiently controlled by SSAs, a condition known to be associated with a more aggressive behavior of the adenoma (Larkin *et al.* 2013).

The second aspect of the study concerns the effect of PEG on the secretion of GH by the pituitary tumor itself. Unexpectedly, we observed a significant and dose-dependent decrease in GH secretion by the tumoral cells when treated by PEG. It appeared as a paradoxical result because the concentration of GH has previously been shown to increase in patients treated with PEG (van der Lely *et al.* 2001, Veldhuis *et al.* 2001). However, in the particular case of GH-secreting primary cultures, the negative IGF1 feedback known to occur *in vivo* (Berelowitz *et al.* 1981) no longer occurs. Yet, IGF1 has been shown to inhibit and accurately regulate the secretion of GH both directly on the somatotroph cells and indirectly through modulation of GHRH and somatostatin release (Berelowitz *et al.* 1981, Gahete *et al.* 2013). Moreover, a previous study showed that addition of IGF1 (100nM) to primary cultures of somatotropinomas indeed leads to a significant decrease of GH secretion in 5/8 tumors, while increasing the PRL secretion of prolactinomas (Atkin *et al.* 1994). In our cultured somatotroph cells, PEG decreased the phosphorylation of JAK2 and STAT5, which suggests a certain degree of basal activation of the GHR by the endogenous GH. The dose-dependent inhibition of GH secretion under PEG further supports the concept of an autocrine/paracrine pattern of action via the GHR and that this activation probably participates in the synthesis and release of GH by the somatotroph, as it has been reported in other models (Zhou et al. 2004a, b). Moreover, we found a trend for a positive correlation between GH inhibition and the GHR mRNA expression. However, it was not possible to assess the impact of the 20-kDa isoform of GH (which would not interfere with the measurement of GH) on the secretion of the 22-kDa isoform because the latter was released within the supernatant and is known to bind to GHR with a better affinity than the 20-kDa isoform (Sigel *et al.* 1981, Smal *et al.* 1986).

The concept of autocrine production of GH through GHR activation (and JAK2/MAPK signaling pathway activation) has already been suggested by Zhou *et al*. (2004*b*) and more recently by the group of Melmed, who showed that STAT3 upregulation in pituitary somatotroph cells led to GH hypersecretion which in turn promoted STAT3 expression (Zhou *et al.* 2015). Interestingly, the same group demonstrated that the GH gene was a transcriptional target for p53 in the pituitary and that p53 activation was correlated with an increase in GH gene transcription as well as GH secretion (Chesnokova *et al.* 2013). Whether p53 is activated by STAT3/5 within the GH cells remains elusive today; however, STAT transcription factors have been shown to activate the p53 pathway in several other experimental models (Chapman *et al.* 2000, Wittig & Groner 2005, Pencik *et al.* 2015). In our model, one hypothesis could be that PEG leads to inhibition of GH secretion by decreasing STAT phosphorylation downstream of the GHR and by the inhibition of p53 activation. Complementary investigations are obviously required to clarify such a hypothesis. Finally, in our experiment, we did not observe any additivity on the inhibition of GH secretion when the PEG and octreotide were used in combination. We hypothesize that because octreotide results in an inhibition of GH secretion in our primary cultures, the GHR is not activated anymore by the autocrine loop and consequently the inhibition effect of PEG no longer occurs.

Besides inhibition of GH secretion, PEG led to a significant inhibition of PRL secretion in the primary cultures of all the three mixed GH/PRL adenomas tested. To our knowledge, there are currently no reports in the literature looking at PRL levels in patients treated with PEG and diagnosed with mixed GH/PRL adenomas. *In vitro*, PRL can inhibit its own secretion by binding its own receptor PRLR in an autocrine/paracrine manner both in humans and in rat pituitary glands extract (Melmed *et al.* 1980, Kadowaki *et al.* 1984). Because PEG does not bind to the PRLR (Goffin *et al.* 1999), it is unlikely that PEG-induced PRL inhibition involves the PRLR. However, a more suitable hypothesis is that PEG actually inhibits simultaneously cosecretion of GH and PRL given the fact that those two hormones coexist within the same secretory granule in the somatolactotroph cell (Bassetti *et al.* 1986). This would imply that molecular pathways triggered by PEG and responsible for PRL inhibition are more or less the same as those involved in GH inhibition, assuming that inhibition of both GH and PRL is due to a blockade in secretory granule exocytosis. Another hypothesis would be that the decrease in PRL observed may be the direct consequence of the GH inhibition in a system in which GH would be a stimulatory hormone for the neighboring lactotroph; however, there are currently no evidences for such a functional interaction. Nonetheless, this appears unlikely because of normal prolactinemia plasma levels observed in certain acromegalic patients despite really high GH plasma levels.

To the best of our knowledge, our work is the first that has focused on the *in vitro* effect of PEG on the growth and hormonal secretion of GH-secreting pituitary tumors. Clearly, PEG has no proliferative impact on human GH-secreting tumor cells, in keeping with the observations made in clinical studies. Moreover, we demonstrate for the first time that *in vitro* PEG inhibits GH secretion of human somatotroph adenomas and PRL release of mixed GH/PRL adenomas. Challenging perspectives arise from this work, especially concerning the intricate mechanisms involved in the effect of PEG that still need to be clarified.

## Declaration of interest

This study was partially supported by an unrestricted research grant from Pfizer.

## Funding

This work was supported by an unrestricted research grant from Pfizer, Centre National de la Recherche Scientifique (CNRS UMR 7286), Aix Marseille University. Caroline Zeiller was the recipient of a fellowship supported by Pfizer through a grant to Protisvalor (Aix Marseille University). Thomas Cuny was the recipient of a fellowship supported by ITMO Cancer INSERM “soutien pour la formation à la recherche translationnelle en cancérologie” 2013 and a fellowship supported by SFE (Société Française d’Endocrinologie) 2012.
